# Hemostatic management of patients undergoing ear-nose-throat surgery

**DOI:** 10.3205/cto000122

**Published:** 2015-12-22

**Authors:** Thomas Thiele, Holger Kaftan, Werner Hosemann, Andreas Greinacher

**Affiliations:** 1 Institute for Immunology and Transfusion Medicine, Section of Transfusion Medicine, University Medicine of Greifswald, Germany; 2 Department of Otolaryngology, Head & Neck Surgery, University Medicine of Greifswald, Germany

**Keywords:** anticoagulation, platelet inhibition, thromboprophylaxis, bleeding risk, thrombosis risk

## Abstract

Perioperative hemostatic management is increasingly important in the field of otolaryngology. This review summarizes the key elements of perioperative risk stratification, thromboprophylaxis and therapies for bridging of antithrombotic treatment. It gives practical advice based on the current literature with focus on patients undergoing ENT surgery.

## 1 Introduction

Perioperative hemostatic management is a significant aspect in otolaryngology that becomes increasingly important. Especially in an aging population there is a major part of patients who have drug induced coagulation abnormalities that require specific strategies before, during, and after surgery to minimize the risk of bleeding or thromboembolic complications. Beside standard post-operative thromboprophylaxis, a growing proportion of patients have an indication for therapeutic long-term anticoagulation and/or platelet inhibition. This is especially challenging because bleeding as well as thrombotic complications need to be prevented effectively. Another problem are patients who present with bleeding complications such as epistaxis under antithrombotic therapy.

For preoperative hemostatic management, the body of evidence with reference to otolaryngology is still limited [1]. The approval of several new anticoagulants and platelet inhibitors during the last years makes perioperative management even more complex. All strategies have to be balanced between the individual patient risks including the urgency of the intervention and the surgical bleeding risk. Pharmacologic characteristics of antiplatelet and anticoagulant drugs can hereby guide management, especially if the available evidence from clinical studies is low. Particularly high risk patients require an interdisciplinary, and often individualized approach for the most appropriate perioperative hemostatic management. 

This review summarizes the current literature and gives a practical advice to perform hemostatic management including risk assessment, thromboprophylaxis, and bridging therapy during ENT surgery.

## 2 Preoperative risk assessment

Before each surgical intervention, the risk of bleeding and thrombosis should be determined by assessing the patients’ history [2] and the risks associated with the planned surgical procedure. Laboratory coagulation tests may also be obtained in special situations but they have a lower negative predictive value than a standardized patient history to identify patients at increased risk for bleeding. 

### 2.1 Bleeding risk

#### 2.1.1 Standardized bleeding history

A systematic preoperative bleeding history is useful to identify patients at risk for peri-procedural bleeding. For this purpose, a validated and standardized questionnaire [2], similar to a checklist, is widely applied. The questionnaire should contain a bleeding history of previous surgery or trauma, a family history, and a detailed history of medication, especially of antithrombotic drug intake [3] including the type of antithrombotic drug. Importantly, some frequently used drugs increase the risk of bleeding such as non-steroidal anti-inflammatory drugs, anti-epileptic drugs, or selective serotonin reuptake inhibitors. They all inhibit platelet function and can substantially aggravate the perioperative bleeding risk in a clinically symptomatic patient, especially if the bleeding symptoms had already been present before these drugs were started.

A negative standardized history has a high negative predictive value and excludes patients with a high risk of bleeding and even laboratory coagulation tests can be omitted in this case [3], [4]. In contrast, a positive standardized history for bleeding needs further assessment including an extended panel of laboratory tests. 

For example, the questionnaires for adults and children applied at our institution are available under: http://www.medizin.uni-greifswald.de/transfus/index.php?id=391.

#### 2.1.2 Preoperative laboratory tests

Platelet count, international normalized ratio (INR) and the activated partial thromboplastin time (aPTT) are routinely performed laboratory tests before surgery to identify patients at risk for bleeding. Importantly, these tests do not assess platelet function, Factor XIII activity and von Willebrand’s factor. Especially platelet function disorders and von Willebrand’s disease are relatively frequent and were found to increase the risk of bleeding after pediatric adeno-tonsillectomy [5]. Furthermore, risk factors for post-tonsillectomy hemorrhage in older patients were mean arterial blood pressure, older age, a history of chronic tonsillitis, and excessive intraoperative blood loss [6] that cannot be assessed by a laboratory workup. Hence, unselective screening of platelet count, INR and aPTT is widely concluded not to be sufficient to identify patients at a higher bleeding risk before ENT surgery [7], [8], [9], [10]. It is a safer procedure to assess the standardized history of patients with bleeding risk by means of a checklist.

Instead, a positive bleeding history should qualify for an extended preoperative coagulation testing [11]. This should include INR, aPTT, von Willebrand’s factor (antigen and activity), factor XIII, thrombelastometry (to rule out hyperfibrinolysis) and a platelet function test. The platelet function analyzer (PFA-100) is sensitive to detect von Willebrand’s disease and can also detect some platelet function disorders. However, it is not sensitive enough to rule out all platelet function defects in a patient with a bleeding history. In case of a positive bleeding history platelet aggregometry should be used to exclude a platelet function defect.

Nevertheless, we suggest obtaining preoperative coagulation tests before surgical interventions before ENT surgery with a high bleeding risk and in patients with expected transfusion requirements including INR, aPTT, platelet count, and fibrinogen [11]. This is helpful to assess the dynamic of coagulation factor consumption and potential development of coagulopathy during or after an intervention and will help to guide the transfusion regimen. Any unexplained deviations of standard clotting assays before surgery should be further clarified because they may indicate rare coagulopathies with an increased bleeding risk [12].

Frequently, patients presenting to an ENT department with bleeding receive antithrombotic treatment. Importantly, antithrombotic drugs influence clotting assays very differently, which makes a careful history very important especially in emergencies [13]. Antiplatelet drugs do not change the INR, aPTT, or platelet counts. In contrast, the INR allows estimating the anticoagulant effect of vitamin K antagonists. 

The new oral direct FXa and FIIa inhibitors affect the INR and aPTT very differently [13], depending on the drug and the reagents used in the laboratory. The thrombin time allows the sensitive detection of the FIIa inhibitor dabigatran, which can also be quantified by the diluted thrombin time. On the other hand anti-FXa assays detect low molecular weight heparins, fondaparinux and the direct oral FXa-inhibitors apixaban, and rivaroxaban [14]. They give quantitative plasma levels of these anticoagulants and are helpful to decide, whether a watch-and-wait strategy until elimination of these drugs is feasible or if any procoagulant therapy should be initiated (Table 5). This is especially relevant in patients admitted with active bleeding or need for urgent surgery. 

#### 2.1.3 Assessment of the surgical bleeding risk

Preoperative estimation of the surgical bleeding risk for ENT patients is mainly based on the experience of the surgeon in each center. To date, no standardized risk assessment has been validated to determine the bleeding risk of each intervention in ENT surgery. Table 1 (Tab. 1) provides an estimate of the bleeding risk in frequent ENT interventions. It is based on our own clinical experience and we apply these dates to balance the risks of different peri-surgical strategies in patients under antithrombotic medication requiring ENT surgery [15], [16]. Determinants for the intraoperative bleeding risk include the extent of tissue damage, location (close to nerves, main blood vessels or important organs) and tissue perfusion. Wound condition after surgery (open/closed wound conditions), tissue damage and the consequences of postoperative bleeding determine the postoperative bleeding risk [17]. These risks may be adopted and changed according to the local experience of each center. 

As a general rule, any use of anticoagulants (beside thrombosis prophylaxes stopped 12 hours before surgery) or antiplatelet medication will lead to a shift towards a higher bleeding risk. Hence, a low risk in patients without anticoagulation may transfer at least into a moderate risk, if patients are treated with antithrombotic drugs. 

#### 2.1.4 Algorithm to identify a bleeding risk

**Figure 1:**
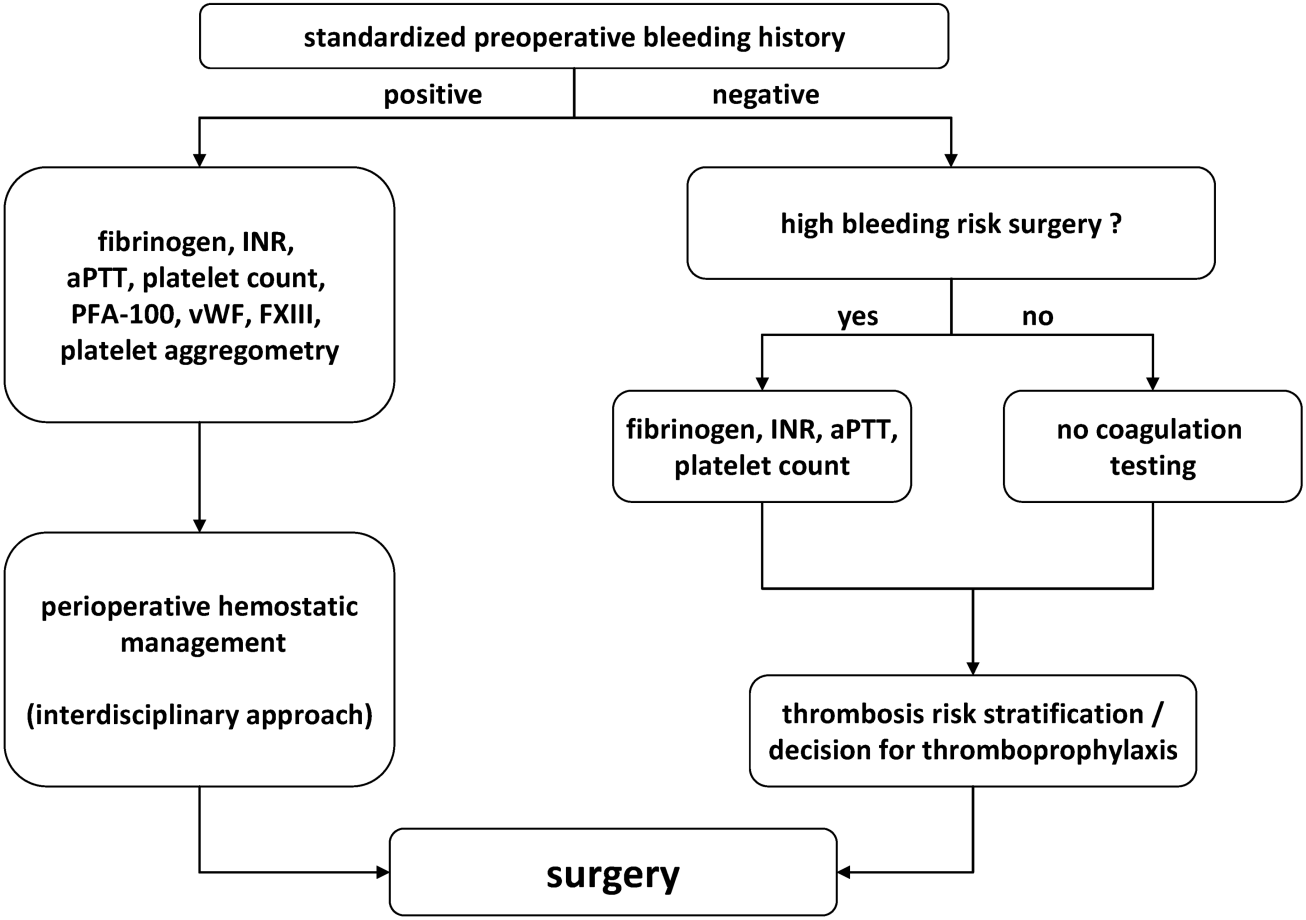
Preoperative algorithm to exclude a bleeding risk

An algorithm to identify ENT patients with an increased bleeding risk is summarized in Figure 1 (Fig. 1). Low-risk patients with a negative bleeding history who require surgery with a low to moderate bleeding risk can undergo surgery without further laboratory assessment. Only if the risk of perioperative bleeding is high, a small panel of laboratory tests might be performed. Thromboprophylaxis should be scheduled if indicated (see section 2.2). 

A positive history for bleeding should prompt further diagnostic work-up. The perioperative management should then be planned between surgeons, anesthesiologists, and hemostaseologists.

### 2.2 Risk of venous thromboembolism

The perioperative risk of venous thromboembolism in ENT patients is relatively low compared to high risk procedures such as orthopedic or abdominal cancer surgery. Most estimates are based on retrospective observational studies and indicate an overall thromboembolic risk below 1% for ENT patients [15], [16]. However, this risks increases considerably in tumor patients [18]. A recent prospective study found up to 13% of ENT tumor patients developing VTE after head neck surgery without routine thromboprophylaxis [19]. This study used ultrasonography to diagnose VTE and included also asymptomatic thrombosis for which the clinical relevance is debated. The incidence of clinically relevant VTE in tumor surgery is likely lower in the range of approximately 6% [20].

Risk factors for the development of VTE can be stratified by a scoring system introduced by Caprini et al. [21] assessing about 40 risk factors. High Caprini scores were also found to be associated with higher VTE rates in ENT patients [22]. However, the application of this scoring system takes time and is therefore not broadly applied in clinical practice. Important risk factors are a history of thrombosis, active cancer, higher age (>60), or planned major surgery (>45min). Also a positive family history of thrombosis in first degree relatives increases the thrombotic risk. Hence, we suggest providing pharmacologic thrombosis prophylaxis for patients with at least one of these risk factors after surgery.

### 2.3 Prothrombotic risk of patients receiving anticoagulants

The most frequent indication for therapeutic dose anticoagulation in elderly patients is prevention of stroke in atrial fibrillation (AF). Temporary interruption of anticoagulation leads to stroke, MI, or systemic embolism in up to 0.7% of AF patients undergoing surgery in the ROCKET-AF trial [23]. However, the risk for a thrombotic complication is primarily enhanced in the period after surgery. For patients with atrial fibrillation the risk of stroke can be estimated with the CHADS_2_-scoring system (Table 2 (Tab. 2)) [24], [25].

A substantially higher prothrombotic risk is observed in patients after heart valve replacement. Here, the type and position of mechanical valves determine the prothrombotic risk. Whereas mechanical mitral valves or replacement of multiple valves have a high risk of thromboembolism, this risk is lower for aortic valves [26]. Furthermore, biological valves have a lower prothrombotic risk. In general it is strongly recommended to consult a cardiologist or expert in hemostasis for perioperative management of patients with heart valve replacement.

For patients receiving therapeutic dose anticoagulation for VTE and/or PE, the time interval between surgery and the last thrombotic event mainly determines the prothrombotic risk. A risk stratification for anticoagulated patients is summarized in Table 3 (Tab. 3) describing a high (>10% per year), moderate (5–10% per year), and low (<5% per year) prothrombotic risk [27].

### 2.4 Prothrombotic risk of patients receiving antiplatelet drugs

Antiplatelet drugs are prescribed for prevention and treatment of arterial occlusions, most importantly for secondary prevention in coronary heart disease. The risk of perioperative arterial thrombosis rises if stents were implanted or if a thrombotic event has occurred closely before surgery. It is important to differentiate drug eluting stents (DES) from bare metal stents (BMS) because the risk of in-stent thrombosis persists longer after DES- than after BMS-insertion. Everolimus eluting stents may be considered to have a lower risk [28], [29] and the recently introduced zotarolimus stents may only require dual antiplatelet therapy for a period of three months [30] after elective stenting. 

Recently, the major adverse cardiac and cerebrovascular event (MACCE) risk criteria were introduced for risk assessment, which distinguishes between high, moderate, and low risk for arterial occlusion [31] (Table 4 (Tab. 4)).

## 3 Perioperative management

### 3.1 Prevention of venous thromboembolism

Options to prevent perioperative venous thromboembolism (VTE) include early mobilization, use of compression stockings, intermittent pneumatic compression, and anticoagulants. The decision, which strategy should be applied depends on patient-specific risks. The risk of bleeding associated with thrombosis prophylaxis is very low, if stopped at least 12 hours before surgery and restarted 6 hours after surgery at the earliest. Current guidelines suggest using thromboprophylaxis only in the presence of strong risk factors such as a positive history of thrombosis, cancer or highly invasive surgery [32], [33]. We propose that concomitant medical illness, higher age and immobility should also qualify for low molecular weight heparin (LMWH) thromboprophylaxis. Tumor patients with extended surgery may receive extended postoperative thromboprophylaxis for at least four weeks because of a persistent strong prothrombotic risk factor [34].

### 3.2 Perioperative management of anticoagulation

#### 3.2.1 Pharmacology and reversal of anticoagulants

Table 5 (Tab. 5) lists oral and parenteral anticoagulants, their pharmacological profile and potential agents to improve hemostasis in emergencies. Hereby it needs to be considered that in patients with renal impairment the half-life of some anticoagulants prolongs substantially even resulting in accumulation of the drug (e.g. LMWH, fondaparinux, danaparoid or dabigatran). 

Reversal of anticoagulants may be required in emergencies. Vitamin-K antagonists (VKAs) can be reversed using prothrombin complex concentrates (PCCs) [35]. This facilitates a rapid improvement of hemostasis before surgery and improves bleeding symptoms. Transfusion of PCCs should be combined with vitamin K application (10 mg i.v.) because of the shorter half-life of vitamin K-dependent clotting factors compared to VKAs. When vitamin K is coadministered with PPCs, endogenous vitamin K-dependent clotting factors can be produced in parallel to the decrease of the transfused clotting factors. 

The only other anticoagulants which can be directly reversed are heparin and in part LMWHs, where protamine can antagonize the anticoagulant effects. Protamin should also be given with caution because it accelerates the bleeding risk again when given at higher doses.

For the other anticoagulants, no specific antidotes are currently available. Prothrombin complex concentrates, activated prothrombin complex concentrates (FEIBA) and recombinant FVIIa have been proposed to overcome severe bleeding episodes [36], [37], but definite clinical data about their efficacy are scarce. Dabigatran can be removed from the blood by dialysis [38], [39] or hemofiltration but these procedures take time and are difficult to perform in emergency situations [40]. 

Currently, specific antidotes are being developed and tested in early phase clinical trials for FIIa- and FXa-inhibitors [41], [42] and will hopefully be available in the next future.

#### 3.2.2 Bridging of VKAs

The term of bridging was developed for VKAs and means to interrupt therapy with a long acting anticoagulant and to switch to a short acting anticoagulant (mainly heparin) which can be stopped shortly before surgery in order to achieve peri-surgery hemostasis. Table 6 (Tab. 6) summarizes the key points of VKA-bridging for each possible risk constellation after the risk assessment described above. All possible risk constellations (Table 3 (Tab. 3)) will be taken into account.

VKAs may not be stopped before procedures with a low bleeding risk in patients with a high risk of thrombosis. Typical low-risk procedures are dermatological interventions or tooth extraction, where the risk of bleeding is increased [43] but can be well controlled. Even pacemaker insertion has a better outcome if anticoagulation with VKAs is maintained instead of interruption and bridging with LMWH [44]. Translated into the ENT setting, this concerns the surgery of skin lesions. Any other ENT intervention even considered to have a low risk of bleeding (Table 1 (Tab. 1)) may be associated with a considerable higher bleeding risk in anticoagulated patients. As bleeding often compromises the postoperative course, we propose to interrupt anticoagulation before most ENT interventions, especially in elective surgery.

Importantly, if a transient high risk of thrombosis exists, surgery should be postponed whenever possible to a time point where the prothrombotic risk has decreased. For example, any elective surgery should be avoided during the first three months after acute thrombosis. If a delay of surgery is not feasible, e.g. if a mechanical mitral valve has been implanted or in emergencies where surgery is immediately necessary, an interdisciplinary coagulation management should be determined to balance patient- and surgery-related risk factors. Those patients may be treated perioperatively with aPTT-adjusted i.v. UFH.

In contrast, for patients with a moderate risk of thrombosis and a high or moderate bleeding risk, therapeutic dose LMWH is not necessary for bridging and VKAs can be interrupted 7 days before surgery. Those patients require (only) preoperative thromboprophylaxis with LMWH if the INR is <2 with the last dose given 12–24 h before surgery. Postoperatively prophylactic dose LMWH can be restarted 6 h post-surgery at the earliest and therapeutic dose anticoagulation can be resumed when the postoperative bleeding risk is low. As vitamin K antagonists require typically 5 days to reach therapeutic INR levels, they can be restarted on the first or second postoperative day.

Patients with a low risk of thrombosis should interrupt VKAs 7 days before surgery without bridging and begin with prophylactic dose LMWH 6–24 h after surgery, depending on the postoperative bleeding risk.

For emergency management of bleeding or urgent surgery, rapid reversal of VKAs with PCCs and vitamin K is possible (see section 3.2.1, Table 5 (Tab. 5)). After reversal and/or surgery, the prothrombotic risks rises again. Thus we suggest starting thromboprophylaxis 24 h post-surgery in the absence of relevant bleeding symptoms.

#### 3.2.3 Management of oral FIIa- and FXa-inhibitors

Elective patients receiving direct oral FIIa- and FXa-inhibitors do not require preoperative bridging therapy because of the shorter half-lives of these drugs (Table 5 (Tab. 5)). The interruption of oral FIIa- and FXa-inhibitors 24 hours before surgery with a low bleeding risk and 48 h before high risk surgery is adequate in most circumstances. Notably, patients with renal impairment may need more time until clearance of the anticoagulant. For dabigatran up to 96 h cessation may be necessary before surgery with a high risk of bleeding [37]. Therefore the renal function should be checked before surgery to exclude a possible accumulation. In this situation laboratory assessment of and direct oral FIIa- and FXa-inhibitor levels may be helpful (see section 3.1.2).

In emergencies, the management of oral FIIa- and FXa-inhibitors depends on the urgency of surgery, the bleeding tendency, and the pharmacokinetic of the involved drug. Most often, a watch-and-wait strategy after stopping oral FIIa- and FXa-inhibitors and conservative treatment of bleedings is possible. This allows the clearance of the drug before surgery and/or resolves bleeding symptoms, respectively. With these drugs, each hour delay in surgery reduces the bleeding risk.

If surgery cannot be delayed, bypassing agents such as activated PCCs, PCCs or recombinant activated factor VII concentrates can be used to improve hemostasis (Table 5 (Tab. 5)). 

Following surgery, the prothrombotic risks rises again and thromboprophylaxis should be started also after 24 h post-surgery, if no relevant bleeding symptoms have occurred. An important aspect of the treatment with oral FIIa- and FXa-inhibitors is their fast onset of action which establishes therapeutic anticoagulation very rapidly. This creates a higher bleeding risk in the postoperative period. Therefore oral FIIa- and FXa-inhibitors should be restarted carefully, especially in surgery with a high postoperative bleeding risk. We suggest to restart therapeutic doses of oral FIIa- and FXa-inhibitors seven days after high risk surgery and to use prophylactic dose anticoagulation with LMWH in the meantime. If the risk of bleeding is low, therapeutic doses of oral FIIa- and FXa-inhibitors may be resumed earlier.

### 3.3 Perioperative management of platelet inhibition

#### 3.3.1 Antiplatelet drugs

Table 7 (Tab. 7) lists the pharmacokinetics of available antiplatelet drugs. Most platelet inhibitors bind irreversibly to their target molecules in platelets and only newly synthesized platelets are able to restore platelet function in vivo. Therefore, most platelet inhibitors cause impaired hemostasis for nearly seven days because this time is needed to synthesize new platelets for a complete platelet turnover. If antiplatelet medication can be stopped prior to surgery, it should be interrupted one week in advance. Acetylsalicylic acid (ASA) is an exception here, where only three days are necessary for cessation [45], [46].

For the emergency management the half-lives of platelet inhibitors and their metabolites are important as well as their binding mode (reversible vs. irreversible). Both conditions determine the possibility to achieve hemostasis with platelet transfusions because circulating active metabolites and reversible platelet inhibitors are able to also inhibit transfused platelets [47]. For example, ASA has a half-life of 20–30 min and is therefore cleared after 2 h under most circumstances. Although the active metabolite of clopidogrel also has a short half-life of 30 min [48], [49], clearance of the active metabolite can require 6–8 hours after intake due to interindividual variations in absorption and metabolism [50], [51] (Table 7 (Tab. 7)).

#### 3.3.2 Management of mono antiplatelet therapy

Monotherapy is most often performed for primary or secondary prophylaxis in patients with a low or moderate MACCE risk (Table 6 (Tab. 6)). ASA is predominantly used here, only in some cases ADP-receptor antagonists are applied for monotherapy. For elective surgery, the recently published POISE-2 study revealed that the interruption of ASA is superior to its continuation in patients with moderate or low MACCE-risk under monotherapy. ASA intake did not reduce the composite of myocardial infarction and death but led to significantly more bleeding complications [45]. According to POISE-2, patients on monotherapy should stop ASA three days before surgery and restart seven days after surgery in the absence of bleeding signs. If the MACCE risk is low, ASA should be paused for 7 days because the risk of re-bleeding is also increased with ASA. Patients with a higher MACCE risk may continue ASA earlier, we suggest to restart ASA not earlier than 6 hours after surgery.

If emergency surgery is required, most ENT operations may be performed even after ASA intake. If applicable, procedures inducing fewer tissue trauma should be performed. Because of the short half-life of ASA, platelet concentrates are an option to treat bleeding complications from ~2 h after the last intake if no surgical control of the bleeding is achieved. 

#### 3.3.3 Management of dual antiplatelet therapy

Patients receiving dual antiplatelet therapy have at least a moderate but most often a high MACCE risk. Elective operations should not be performed in high-risk patients and postponed to a time point when the risk is lower (Table 4 (Tab. 4)) [48], [52]. 

For time critical surgery, current cardiology guidelines encourage surgeons to perform surgery without interrupting DAPT in high risk patients [48]. For most urgent ENT procedures the intraoperative bleeding risk is tolerable [49]. In contrast, antiplatelet drugs increase postoperative bleeding complications in non-cardiac surgery and in ENT patients [17], [53], [54] and it is not clear, whether re-bleedings may cause more damage by resulting in more re-interventions with additional risks for these patients. It is also debated, whether an increased bleeding risk even results in higher cardiac morbidity because it may cause circulatory depression which results in lower coronary perfusion [55]. As these questions are not yet answered, surgery in patients with a high MACCE risk under dual antiplatelet therapy needs an interdisciplinary approach involving surgeons, hemostaseologists, anesthesiologists, and cardiologists [52]. 

**Figure 2:**
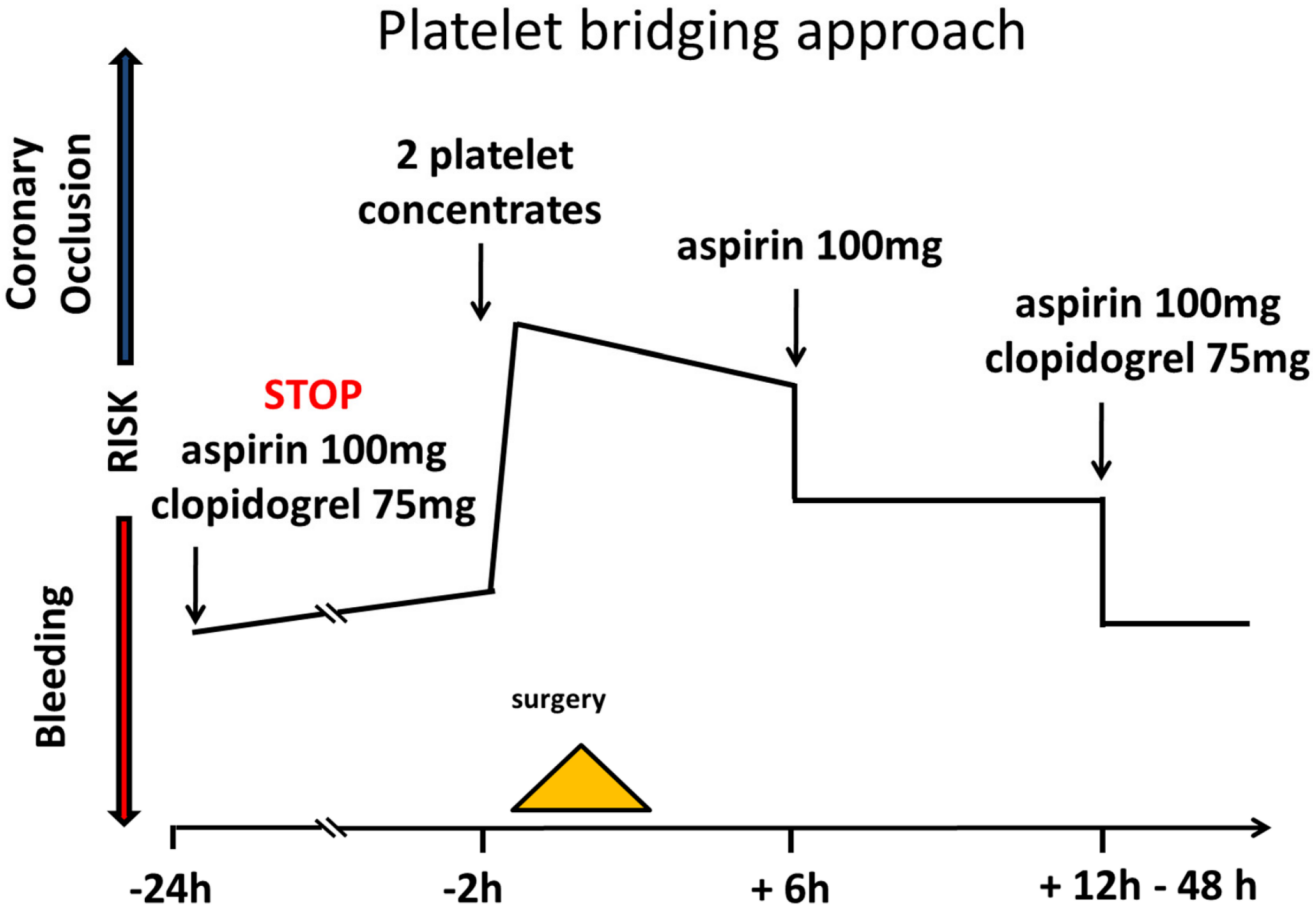
Greifswald bridging protocol for urgent surgery under dual antiplatelet therapy

To achieve surgery in patients with high MACCE risk where surgery cannot be postponed we designed a protocol to ‘antagonize’ antiplatelet therapy using prophylactic platelet transfusions [56]. Based on the pharmacokinetic profile of ASA with a half-life of 30 min and clopidogrel with a persistence of active drug for 6–8 hours, platelet transfusion is scheduled 12–24 hours after last intake of these drugs and surgery is performed subsequently. ASA is restarted 6 to 12 hours after surgery. Only if the patient does not develop increased bleeding after 12 to 24 hours on ASA, clopidogrel is continued (Figure 2 (Fig. 2)). 

This protocol should not be applied indiscriminately to other antiplatelet drugs because of their different pharmacokinetic profile (see above). Active metabolites of prasugrel and ticagrelor have a half-life of 8 hours [57] and 13 hours [58], respectively, with up to 96 hours of increased bleeding risk for ticagrelor. Importantly, patients with a high MACCE risk can develop acute coronary syndrome intraoperatively. Thus, PCI-facilities should be available at centers performing these high risk interventions.

Only few alternatives exist for the perioperative management of patients under dual antiplatelet therapy. Bridging with tirofiban has been suggested [59], but this requires intravenous infusion for several days and underdosing might even increase the risk of coronary events. Treatment with ‘prohemostatic’ drugs such as desmopressin or tranexamic acid could pose significant risks of coronary thrombosis. 

Of note, bridging with heparin does not protect against new coronary thrombosis or myocardial infarction [60] and should therefore not be used to replace antiplatelet drugs perioperatively.

## 4 Concluding remarks

Hemostatic management in ENT surgery is increasingly complex. The higher the risk of bleeding or thrombosis, the more precautions are necessary. Currently only few data are available assessing hemostatic management in ENT patients. Furthermore, for the novel oral FIIa- and FXa-inhibitors specific antidotes will become available which will allow a much better management in emergencies. Beside all statistical measures it should be acknowledged, that especially in high-risk patients an individualized and interdisciplinary approach is necessary to balance patient-specific risk factors. This will remain a key element also in the future to optimize the perioperative hemostasis management.

## Notes

### Competing interests

Relative to anticoagulants and clotting factors concentrates, the authors disclose the following competing interests:

- Thiele: training support Bristol Myers Squibb, Novo Nordisk, Bayer; investigator Boehringer Ingelheim
- Greinacher: consultant Bayer; consultant + investigator Boehringer Ingelheim, Bristol Myers Squibb
- Hosemann: Fa. Karl Storz GmbH, Tuttlingen; Fa. Medtronic, Meerbusch.

## Figures and Tables

**Table 1:**
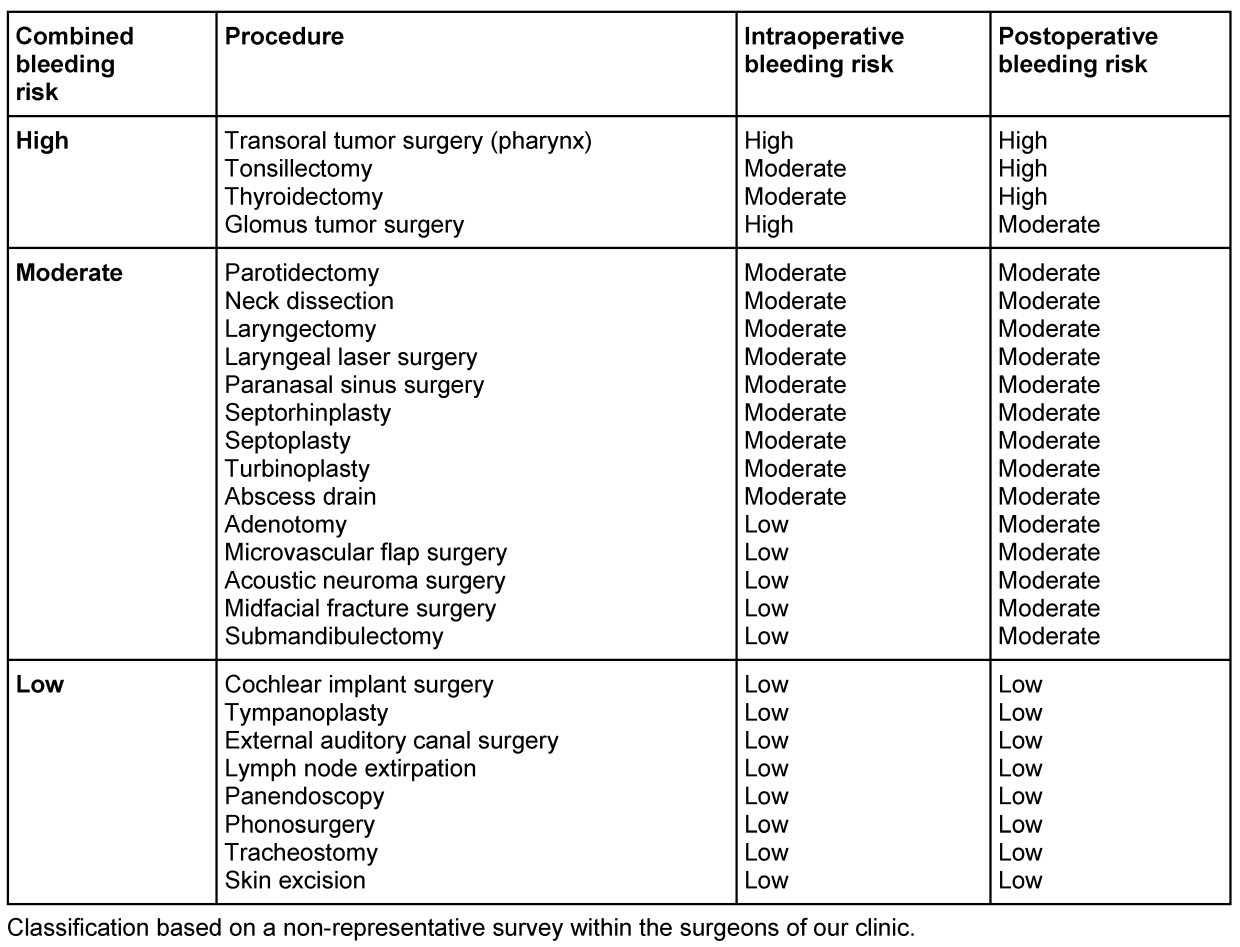
Bleeding risk of frequent ENT operations

**Table 2:**
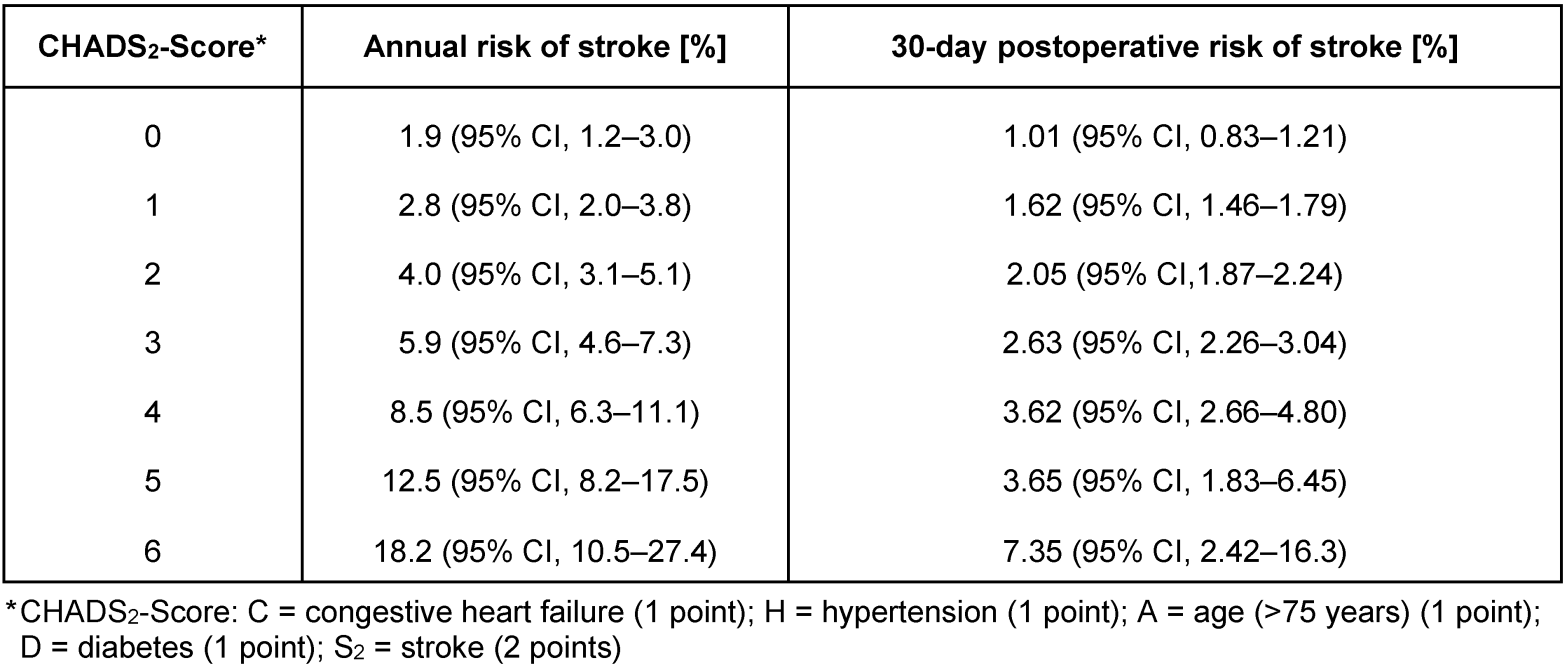
CHADS_2_ Score and the risk of stroke in patients with atrial fibrillation (adapted from [24, 25, 61])

**Table 3:**
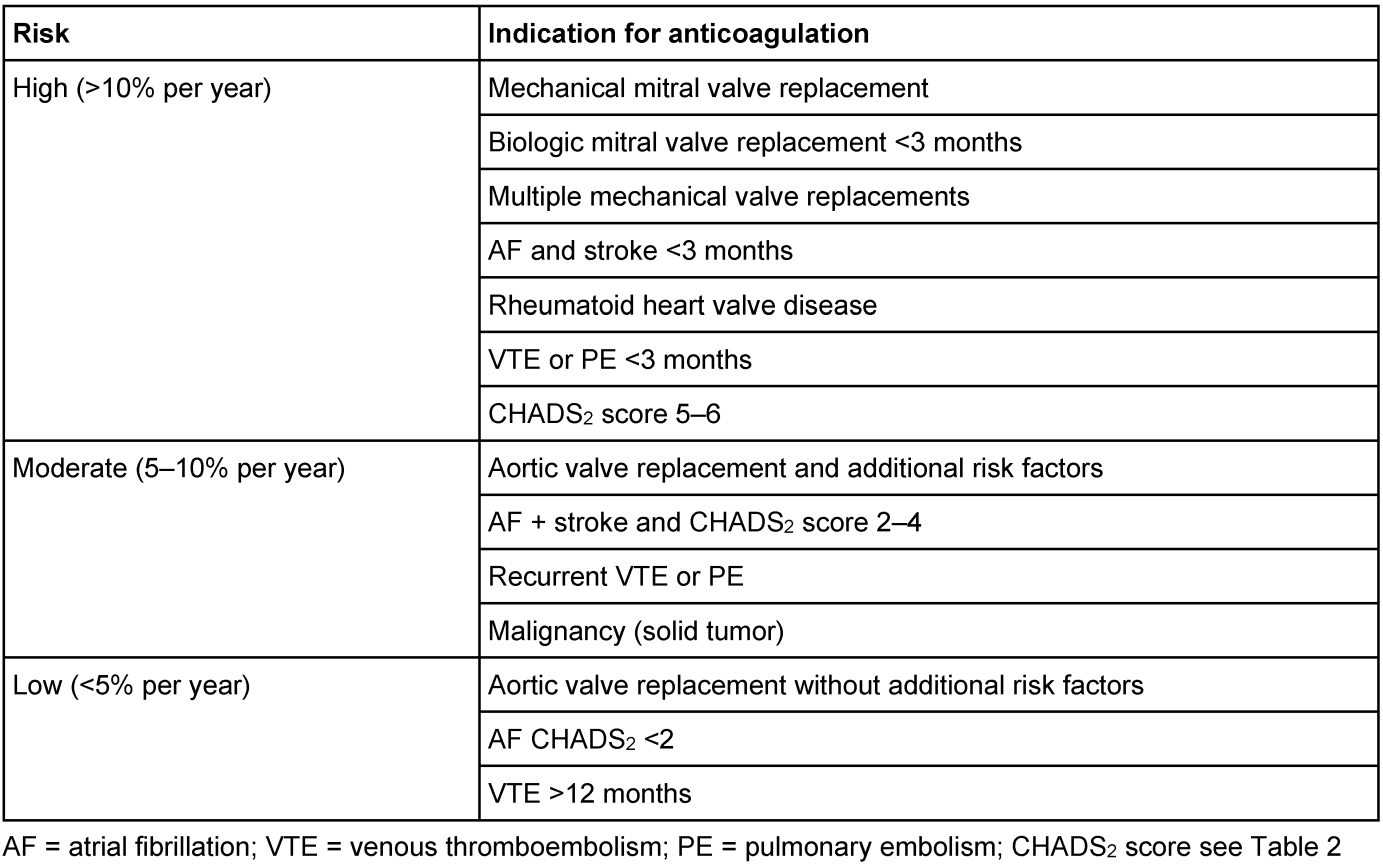
Thrombotic risk of patients under anticoagulation therapy (adapted from [1, 24, 26, 27, 62])

**Table 4:**
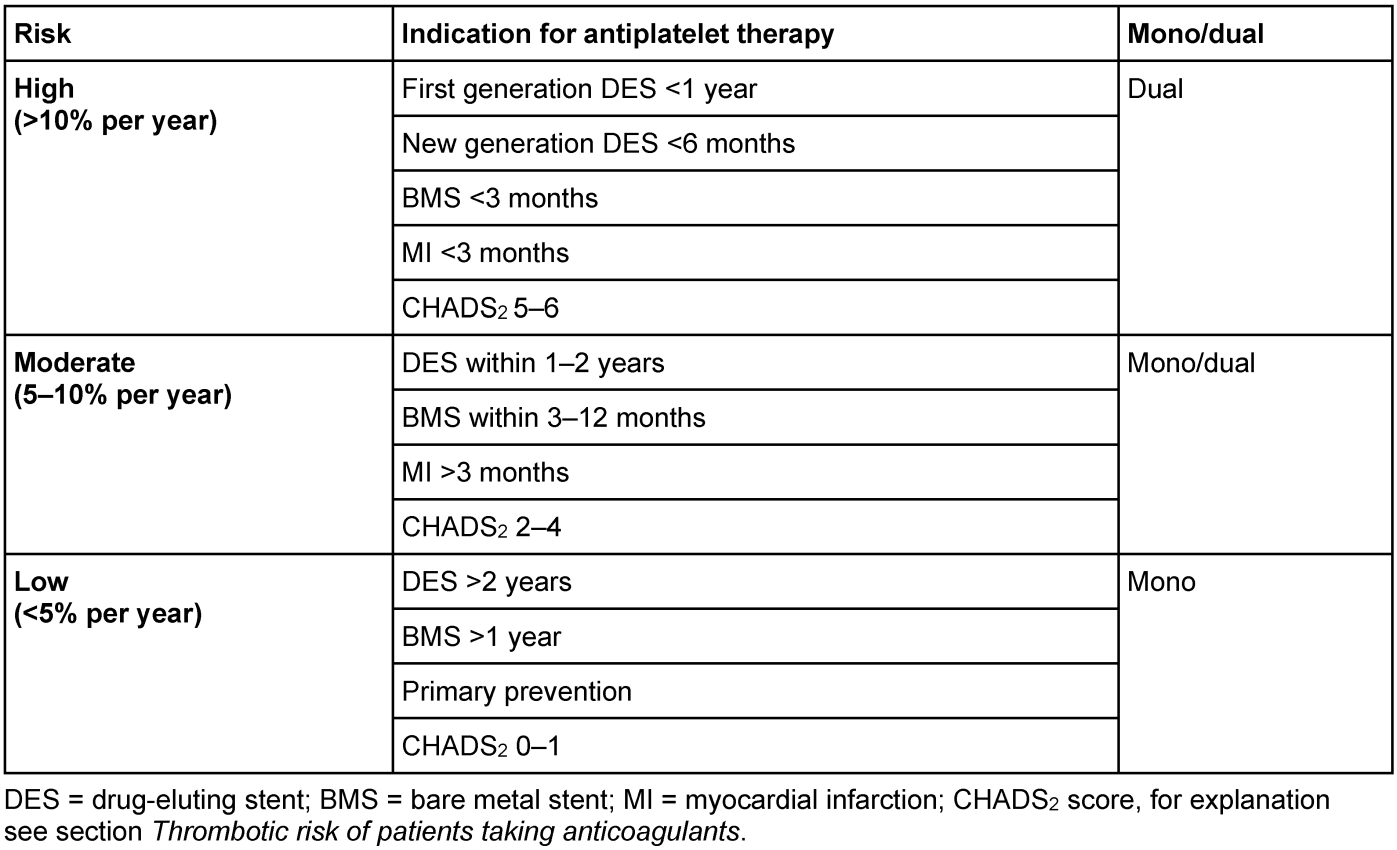
MACCE-risk of patients taking antiplatelet drugs (adapted from [31])

**Table 5:**
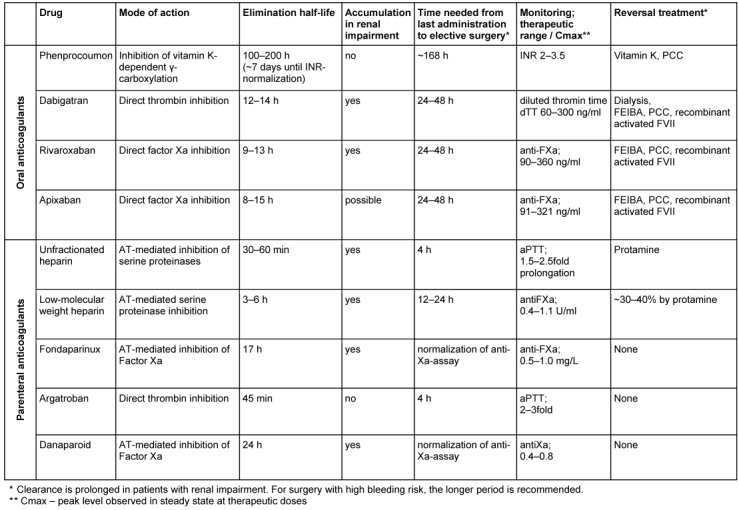
Anticoagulants (adapted from [1, 63] and from the respective prescribing information)

**Table 6:**
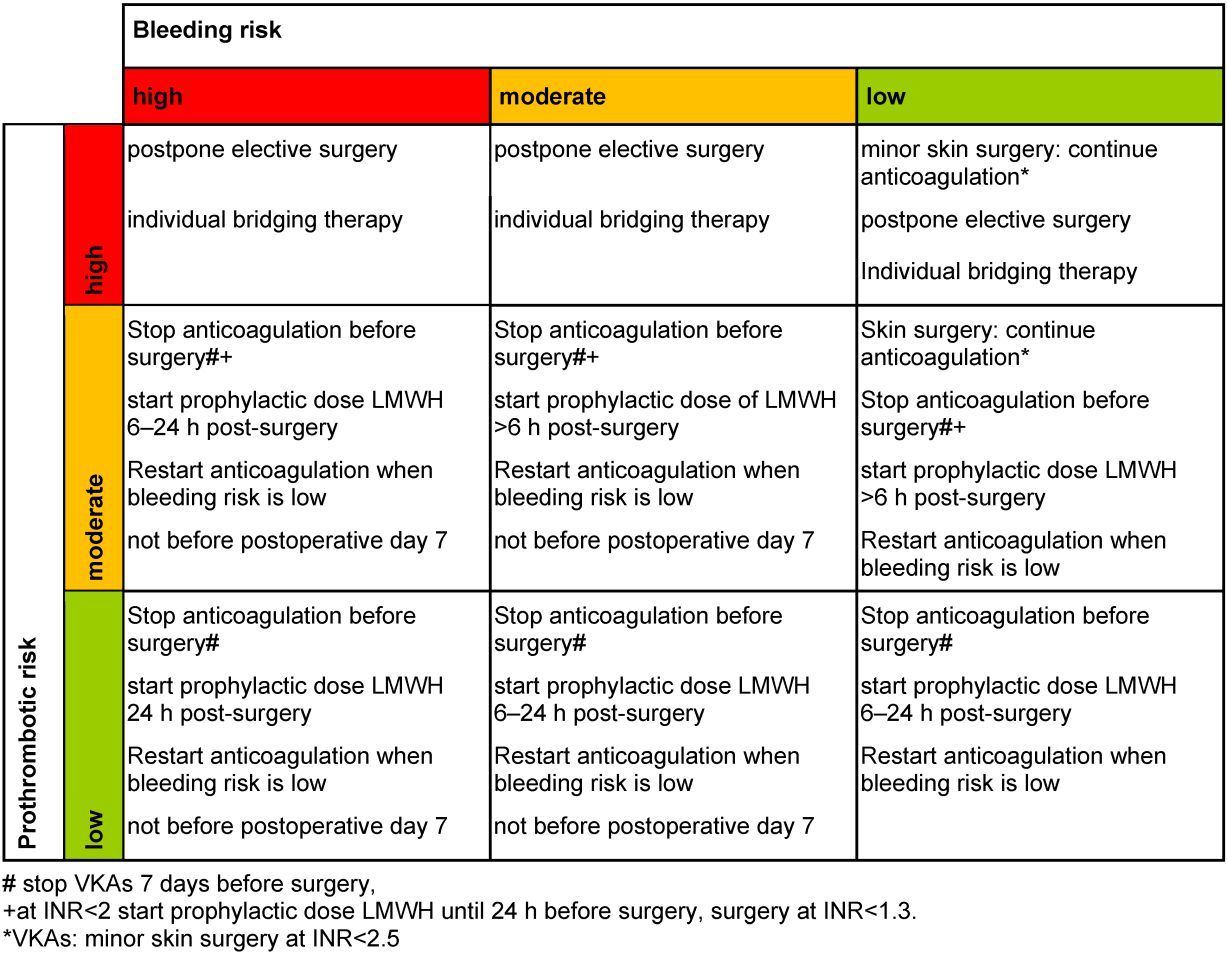
Bridging scheme for VKAs

**Table 7:**
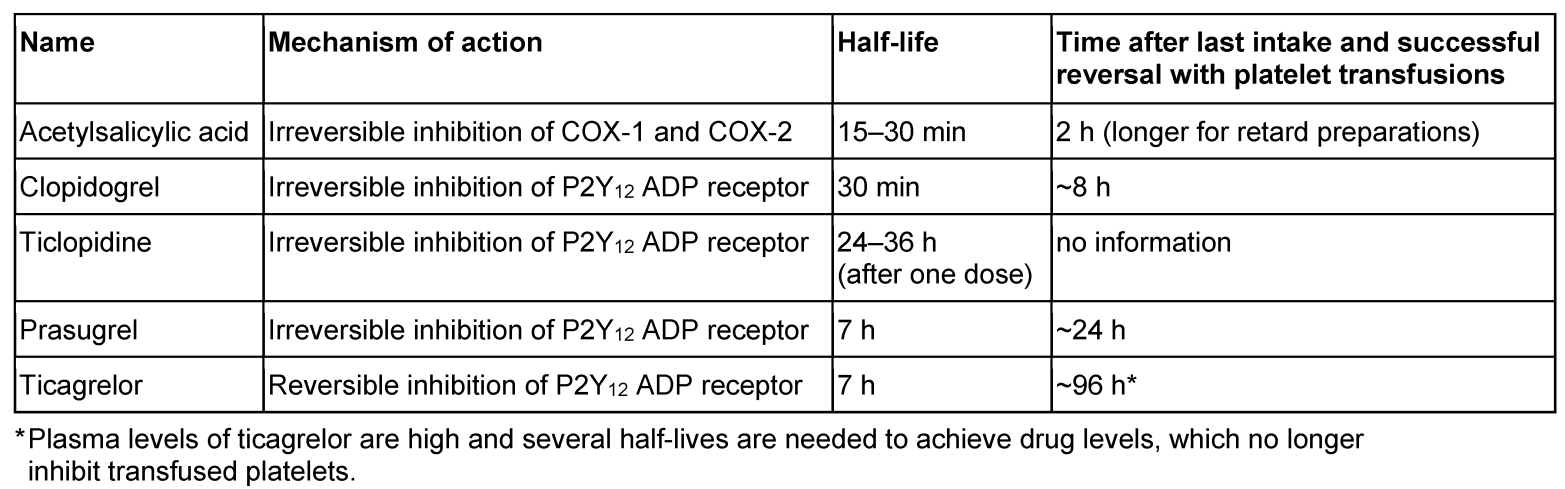
Antiplatelet drugs (adapted from [1] and respective prescribing information)
